# Evaluation of the Oscillatory Interference Model of Grid Cell Firing through Analysis and Measured Period Variance of Some Biological Oscillators

**DOI:** 10.1371/journal.pcbi.1000573

**Published:** 2009-11-20

**Authors:** Eric A. Zilli, Motoharu Yoshida, Babak Tahvildari, Lisa M. Giocomo, Michael E. Hasselmo

**Affiliations:** 1 Department of Psychology, Boston University, Boston, Massachusetts, United States of America; 2 Department of Neurology and Neurosurgery, Montreal Neurological Institute, McGill University, Montreal, Canada; École Normale Supérieure, College de France, CNRS, France

## Abstract

Models of the hexagonally arrayed spatial activity pattern of grid cell firing in the literature generally fall into two main categories: continuous attractor models or oscillatory interference models. Burak and Fiete (2009, PLoS Comput Biol) recently examined noise in two continuous attractor models, but did not consider oscillatory interference models in detail. Here we analyze an oscillatory interference model to examine the effects of noise on its stability and spatial firing properties. We show analytically that the square of the drift in encoded position due to noise is proportional to time and inversely proportional to the number of oscillators. We also show there is a relatively fixed breakdown point, independent of many parameters of the model, past which noise overwhelms the spatial signal. Based on this result, we show that a pair of oscillators are expected to maintain a stable grid for approximately *t = 5µ* ^3^ */(4πσ)* ^2^ seconds where *µ* is the mean period of an oscillator in seconds and σ^2^ its variance in seconds^2^. We apply this criterion to recordings of individual persistent spiking neurons in postsubiculum (dorsal presubiculum) and layers III and V of entorhinal cortex, to subthreshold membrane potential oscillation recordings in layer II stellate cells of medial entorhinal cortex and to values from the literature regarding medial septum theta bursting cells. All oscillators examined have expected stability times far below those seen in experimental recordings of grid cells, suggesting the examined biological oscillators are unfit as a substrate for current implementations of oscillatory interference models. However, oscillatory interference models can tolerate small amounts of noise, suggesting the utility of circuit level effects which might reduce oscillator variability. Further implications for grid cell models are discussed.

## Author Summary

For many animals, including rats, accurate spatial memory over relatively large areas is important in order to find food and shelter. Just as unique points in time can be efficiently represented by combinations of repeating elements like hours, days, and months, points in space can be represented as combinations of elements that repeat at different spatial scales. Just such a code has been identified in the brains of rats and it shows an intriguing triangular spacing of encoded locations. Two different explanations have been developed as to what general mechanism in the brain might be able to generate this unusual code. However, to date there is not conclusive experimental evidence indicating whether either of the two explanations is correct. Here we show in detail that one of the explanations, called oscillatory interference, has specific requirements regarding the amount of variability in the system that implements it. We then report data experimentally examining candidate systems to evaluate their levels of noise. The large amount of noise that we find presents a challenge to the currently suggested biological implementations of oscillatory interference, but it does not provide support for the alternative explanation.

## Introduction

Grid cells are a type of cells first found in rat medial entorhinal cortex [1]–[3] that are characterized by their spatial firing correlates. Each cell has multiple place fields (locations in the environment where the cell fires at a high rate) which are located at the vertices of an equilateral triangular tessellation of the environment.

Two main types of models have been proposed to explain the spatial firing properties of these cells. The first class consists of continuous attractor models [4]–[7] (see [8] for a slightly different approach). These models use complex internal connectivity to form a grid of activity on a sheet of neurons and then use a body velocity signal to slide the grid of activity in register with the animal's movements. The second class consists of oscillatory interference models [9]–[13]. In these models the hexagonal pattern arises from two or more oscillators as an interference pattern. Candidate oscillators initially included oscillations of local field potentials and the membrane potential oscillations of entorhinal cortical layer II stellate cells which occur when the cells are brought just below threshold [11],[14]. Reference [13] implemented an oscillatory interference model using persistent spiking cells. Such cells are found in multiple locations in medial temporal cortex, such as entorhinal cortex and postsubiculum [15]–[18]. Reference [19] suggests the oscillators might be located in subcortical rhythm generating structures. Formally the models are all sufficiently similar that the following results should apply to all of the cited versions.

Reference [7] provided an excellent discussion of many predictions that can differentiate between the two classes of models. We contribute to this discussion by evaluating the suitability of a range of biological oscillators for the oscillatory interference models.

In particular we examine the behavior of an oscillatory interference model when noise is introduced into its oscillators. A common criticism [7],[13],[20],[21] of this class of models is that real neural systems are too noisy to maintain the perfect oscillations that are usually simulated. Noise-related problems might be avoided by having external cues reset the grid network [11],[22],[23], but only if noise levels are low enough (because the grid network must be stable enough that fields are more or less at their correct location during initial exploration for the external cue associations to be made). One group [21] qualitatively examined traces of subthreshold membrane potential oscillations and roughly estimated that such oscillations might provide a stable representation of place for 0.5 seconds. Here we show that, for certain configurations of the model, the model can be surprisingly robust to moderate amounts of noise (for instance, the drift in encoded position due to noise is inversely proportional to the number of oscillators). However, we find a relatively fixed noise threshold past which it is unlikely that a grid cell will still be correctly encoding position.

We then use this noise threshold to derive a relation which can be used to evaluate the noise level in neuronal oscillators. Letting 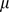 be the measured mean period in seconds of the biological oscillator and 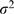 the variance of the period in 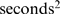, then a biological system can be expected to maintain a stable grid pattern for approximately 
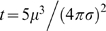
 seconds (the time at which the variance of the cumulative noise reaches the noise threshold).

The candidate oscillators that we examine experimentally are persistent spiking neurons in layers III and V of entorhinal cortex as well as in postsubiculum (dorsal presubiculum) and subthreshold membrane potential oscillations in entorhinal cortex layer II. The former regions all project directly to entorhinal cortex layer II [24] and so are well positioned to be the oscillators in at least one oscillatory interference model [13]. In addition, these regions contain head-direction signals [3],[25] which are sometimes used as a surrogate for body velocity. Further, grid cells have been reported in all of these regions [3],[26], raising the possibility the characteristic spatial pattern is created in one of these regions and simply inherited by downstream neurons. We find that the estimated stability times from the experimental data are on the order of a few seconds, much shorter than the reported stability of grid cells. This suggests the current oscillatory interference models must be modified to account for biological levels of noise, and we suggest one possibility in the Discussion.

## Materials and Methods

### Ethics Statement

Our experiments were performed at two different locations. Experimental protocols were approved by the Institutional Animal Care and Use Committee at Boston University or were approved by the McGill University Animal Care committee and were in compliance with guidelines of the Canadian Council on Animal Care.

### Computational Methods

**Figure 1:**
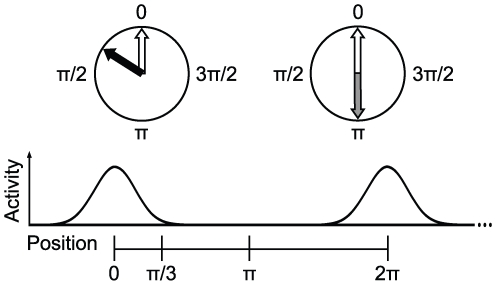
Coding 1D angular position. Top. We can represent oscillators as an arrow spinning on a circle such that the rotational speed of the arrow represents the frequency of the oscillator and the current angle of the arrow represents its phase. Additional oscillators can be represented by additional arrows on the same plot. Bottom. Because different grid cells have different spacings, it is convenient to measure distance between fields in different units, such as angles. Each position between two fields can be uniquely identified by its corresponding angle. Such angles can be encoded as the phase difference between two oscillators with the same frequency. On the left, the arrows encode a phase difference of 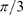. On the right, the arrows encode a phase difference of 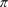.

To introduce the oscillatory interference model, we consider a pair of oscillators represented as spinning arrows, Figure 1, top. We assume that the oscillators have the same frequency (rotational speed of the arrow) but may differ in phase (the direction the arrow is pointing at any given time).

The difference in phase is used to encode a position in space, Figure 1, bottom. Each position along the line connecting neighboring grid fields corresponds to a phase difference. Since the spacing of grid fields varies in cells recorded at different positions along the dorsoventral axis of medial entorhinal cortex [2], it is convenient to set aside physical units and speak of spatial distances directly in terms of the corresponding phase difference. [11],[13] have given the relation between phase differences and spatial distances, which can vary depending on the form of the model, but relate to one or both of 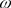, the temporal frequency of the baseline oscillation, and 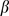, a parameter relating temporal frequencies to spatial frequencies. Parameter values fit to electrophysiologically recorded subthreshold membrane potential oscillations in putative grid cells in entorhinal cortex are given in [14]. Model parameters used in our simulations were appropriate for a dorsally-located grid cell in entorhinal cortex layer II: 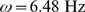, 
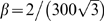
 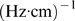 [14].

In this way, the pair of oscillators encodes a position on a circle (angular position) which is mapped onto space. As the animal walks along a line and passes through each grid field, the cell's encoded position moves around a circle back through the same field each time (like the repeating backgrounds in old cartoons).

To encode a position in two dimensions, an additional oscillator is included. One of the oscillators remains a baseline oscillator and the other two independently encode angular position in two directions. These angular position encoding oscillators are called velocity-controlled oscillators (VCOs) for reasons explained below. Each VCO has a preferred direction in space, and the pairwise differences of preferred directions must be multiples of 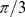 and not all co-linear in order to produce the hexagonal pattern seen in animals. Indeed, arbitrarily many VCOs can be included (see later) as long as they meet these angle requirements.

In order to update the encoded spatial position, the model requires input about body velocity (sometimes equated with a velocity-modulated head direction input). To adjust the encoded angular positions, the VCOs change their frequency as a function of the velocity input (hence the term velocity-controlled oscillators). This is covered in detail in earlier treatments (e.g. [11],[12],[27]). The essential point for the present discussion is that, for noiseless oscillators, the phase differences always perfectly code for the animal's current position.

We close with a formal description of the model. We consider two different activation rules: a rectified product rule (used in most simulations) and a sum rule (to demonstrate that effects described later do not depend on the activation rule). The output of the model with a rectified product activation rule at time 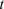 is given by the equation:
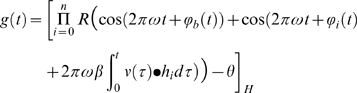
(1)where 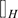 is the Heaviside step function, 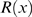 is the ramp or threshold-linear function (to model half-wave rectification), 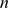 is the number of VCOs, 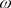 is a baseline frequency, 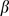 a constant relating temporal frequencies to spatial frequencies, 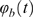 and 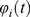 are the phase noise functions described in the next section (which may also include a non-zero starting phase), 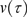 is the vector-valued velocity function of the agent at time 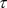, 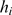 is a unit vector in the preferred direction of VCO 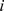, 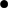 represents a dot product, and 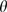 is a firing threshold. Simulations were performed using 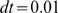 and threshold 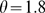.

The half-wave rectification prevents the product of an even number of out-of-phase sinusoids from having a net positive effect on the activity of the cell.

The integral of the dot product of the animal's velocity history with each VCO's preferred direction calculates the total distance travelled in the preferred direction, which is then multiplied by 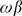 to convert the distance into an angular position which becomes the phase difference between the two oscillators (setting aside the noise terms for now).

The sum rule we use is
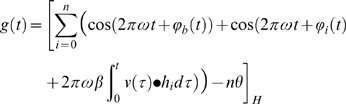
(2)


Which is as (1) but notice here that the firing threshold increases linearly with the number of oscillators and we use a higher value of 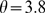.

For the purpose of plotting spatial firing rate maps of the model, it is convenient to calculate the proportion of the time during one baseline oscillation that the cell is above threshold.

We consider accumulating noise that causes the phase differences to drift away from their correct values. There are many noise sources in cells, but the dominant two are stochastic ion channel opening/closing and synaptic noise [28]–[30]. We will be comparing simulation results to experimental results from single neurons recorded in the presence of synaptic blockers, so we will focus only on intrinsic noise sources, of which ion channel fluctuations dominate [28]. Ion channel noise has both additive (voltage-independent) and multiplicative (voltage-dependent) components [28], but the multiplicative components are often ignored for analytic simplicity because of their fast time scales [28],[31]. The oscillators used in current oscillatory interference grid cell models are abstract, sinusoidal functions which simply change their phase at a fixed frequency, so we approximate this channel noise by adding a normally-distributed, zero-mean noise term to the phase of an oscillator on each simulation time step (a common noise model, e.g. [32]). We consider noise both in the VCOs as well as in the baseline oscillation, which introduces covariance in the noise terms for each phase difference.

We write the noise introduced into the baseline oscillator's phase on time step 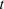 as 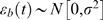 (i.e., 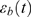 is a random variable drawn from the normal distribution with mean 0 and variance 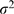). The noise introduced into VCO 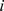 is 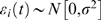 and all of these noise terms are taken to be independent. A non-zero mean would represent a bias in the input that would cause the spatial grid pattern to drift constantly (see Discussion). Since such a drift is not seen in the experimental grid cell recordings, we assume a mean of zero (continuous attractor models require the same assumption). At time 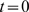, the cumulative noise in a phase difference will be 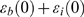. After 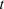 time steps, the cumulative noise will be
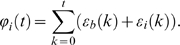
(3)


Each 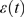 is independently, normally distributed, so 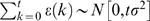 for all noise terms (in statistical terms, 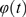 is essentially a discrete analog of a Wiener process). Each phase difference accumulates noise from both the baseline and the velocity-controlled oscillator and so the cumulative noise in a phase difference will be distributed 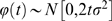. The variance thus increases by 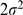 on each time step. Because of the shared baseline noise, the cumulative covariance of the noise between two phase differences will equal 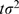 after 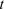 time steps (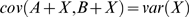 for 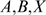 independent random variables). Therefore the cumulative noise covariance matrix 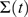 has 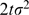 on its diagonal and all other entries equal 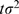.

The normal distribution is defined on the real numbers. However, the noise is being added to an oscillator's phase: a circular variable defined on the interval 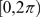. This is important because it means the difference between the encoded phase difference and the correct phase difference is bounded: the difference cannot be 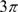, for instance, though a normal distribution might have nonzero density at that point. This means the probability that the cumulative noise is between 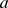 and 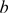 is not simply 
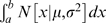
 (where 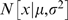 is the normal distribution's probability density function evaluated at 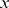), because the cumulative noise would appear to be in the range 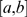 if it were actually in the ranges 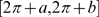 or 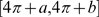 or 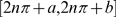 for any integer 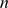. To identify the probability that the cumulative noise is in a specific range requires summing an infinite number of integrals with consecutive lower (and upper) bounds spaced 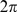 apart. This is called the wrapped normal (WN) distribution and its probability density function (pdf) is
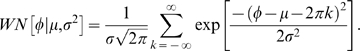
(4)


This allows us to calculate the probability that the noise is in a certain range, given the cumulative noise variance.

To take into account the covariance among the phase differences, we treat them as a vector and use the multivariate wrapped normal distribution (MWN) with nonsingular noise covariance matrix 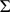:M
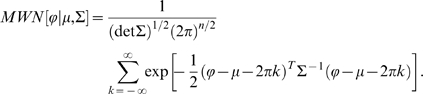
(5)where 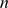 is the number of VCOs.

We will consider the effects of noise both on the individual cell level as well as on the population level. The latter is important because a given animal may have many grid cells with the same orientation, spacing, and phase. Each such cell will drift independently due to noise, which, as we show, allows the population average to be a more reliable signal than individual cells provide.

### Whole-Cell Patch Recording

For postsubiculum slice preparation, Long-Evans rats (postnatal days 21 to 25; Charles River, Wilmington, MA) were deeply anesthetized with ketamine/xylazine (95 mg/Kg ketamine and 2.8 mg/Kg xylazine) through intraperitoneal injection. After the absence of both pedal and tail pinch reflex was confirmed, ice-cold modified artificial cerebrospinal fluid (ACSF) containing (in mM) 110 choline chloride, 2.5 KCl, 1.25 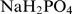, 26 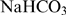, 0.5 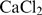, 7 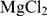, 7 glucose, 3 pyruvic acid and 1 ascorbic acid (pH adjusted to 7.4 by saturation with 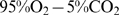) was intracardially perfused. For the entorhinal cortex layer II and III slice preparations, Long-Evans rats (postnatal days 17 to 23; Charles River, Wilmington, MA) were deeply anesthetized with isoflurane (Abbot Laboratories) and decapitated after the absence of both pedal and tail pinch reflex was confirmed.

The brain was then removed from the cranium and placed in ice-cold modified ACSF. 350 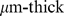 slices were cut sagittally or horizontally using a Vibroslicer (World Precision Instruments, Sarasota, FL, USA). Slices were transferred to a holding chamber, where they were kept submerged at 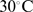 for 30 min and then at room temperature at least 30 min longer before recording. The holding chamber was filled with ACSF containing (in mM) 124 NaCl, 3 KCl, 1.25 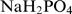, 26 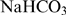, 1.6 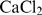, 1.8 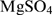, 10 glucose (pH adjusted to 7.4 by saturation with 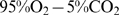).

Entorhinal cortex layer III and postsubiculum slices were transferred to a submerged recording chamber and superfused with ACSF, maintaining the temperature between 34 and 36 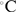 for recordings. Entorhinal cortex layer II slices were transferred to a submerged recording temperature maintained between 36 and 38 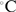. Patch pipettes were fabricated from borosilicate glass capillaries by means of a P-87 horizontal puller (Sutter Instrument, Novato, CA, USA). Patch pipettes were filled with intracellular solution containing (in mM) 120 K-gluconate, 10 HEPES, 0.2 EGTA, 20 KCl, 2 MgCl, 7 phosphocreatine-di(Tris), 4 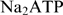 and 0.3 TrisGTP (pH adjusted to 7.3 with KOH). The intracellular solution also contained 0.1% biocytin for the purpose of labeling. When filled with this solution, the patch pipettes had a resistance of 3–5 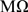. Slices were visualized with an upright microscope (Zeiss Axioskop 2), equipped with a 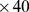 water-immersion objective lens and a near-infrared charge-coupled device (CCD) camera (JAI CV-M50IR, San Jose, CA, USA). Tight seals (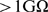) were formed on cell bodies and the membrane was ruptured with negative pressure. Current-clamp recordings were made with a Multi Clamp 700B amplifier (Axon Instruments, Foster City, CA, USA) using a built-in bridge balance and capacitance compensation circuits. Signals were low-pass filtered at 5 kHz or 10 kHz and sampled at 10 kHz or 20 kHz, respectively, using Clampex 9.0 software (Axon Instruments, Foster City, CA, USA). A liquid junction potential of 10 mV was not corrected.

Chemicals were purchased from Sigma-Aldrich (St. Louis, MO, USA) and Tocris Bioscience (Ellisville, MO, USA).

### Sharp Electrode Recording

Procedures of slice preparation and intracellular recording of layers III and V entorhinal neurons were described in detail in [33] and [34], respectively. Conventional sharp microelectrode intracellular recordings were performed on brain slices obtained from adult Long-Evans rats (male, 150–250 g; 4–5 weeks postnatal, Charles River Canada, Saint-Constant, Quebec, Canada) at 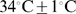 in an interface recording chamber (Fine Scientific Tools, North Vancouver, British Columbia, Canada). Normal Ringer solution was prepared daily and contained (in mM) 124 NaCl, 3 KCl, 1.6 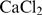, 1.8 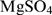, 26 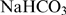, 1.25 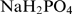, and 10 glucose (pH was adjusted to 7.4 by continuous application of 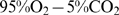). Since the muscarinic phenomena studied did not desensitize, neurons were initially impaled in the presence of carbachol (CCh) (10 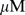). All the chemicals were purchased from Sigma.

### Induction and Measurement of Persistent Firing

Induction of persistent firing was tested in the presence of carbachol (10 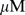) and synaptic blockers including kynurenic acid (2 mM) and picrotoxin (100 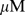) to suppress ionotropic glutamate receptors and 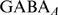 receptors, respectively. Membrane potential was adjusted to just below rheobase membrane potential using constant current injection. After confirming that the baseline potential was not drifting for at least 5 s, a supra-threshold current injection with the duration of 2 to 4 s and the amplitude between 50 to 300 pA was applied to induce persistent firing. Frequency of persistent firing was measured as an average firing frequency of the neuron during a 20 s period starting at 7 to 30 s after the termination of the current injection. Clampfit 9.0 (Axon Instruments) and MATLAB (MathWorks) were used for data analysis.

### Measurement of Membrane Potential Oscillations

These methods have been described in detail previously [20]. Briefly, layer II stellate cells were depolarized to just below action potential firing threshold by applying small current steps. When the cell neared firing threshold and membrane potential oscillations appeared, long segments of membrane potential were recorded. Membrane potential oscillations were analyzed by an automated script in MATLAB to determine the peak-to-peak interval. A Butterworth filter between 0 and 15 Hz was applied and an automated peak detection function (peakdetect) in MATLAB determined the peak-to-peak intervals for oscillations at a membrane potential of 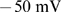.

## Results

The behavior of the model changes as a function of the number of VCOs. We first individually examine the 1 and 2 VCO cases before considering the case for 3 or more VCOs.

### One VCO

**Figure 2:**
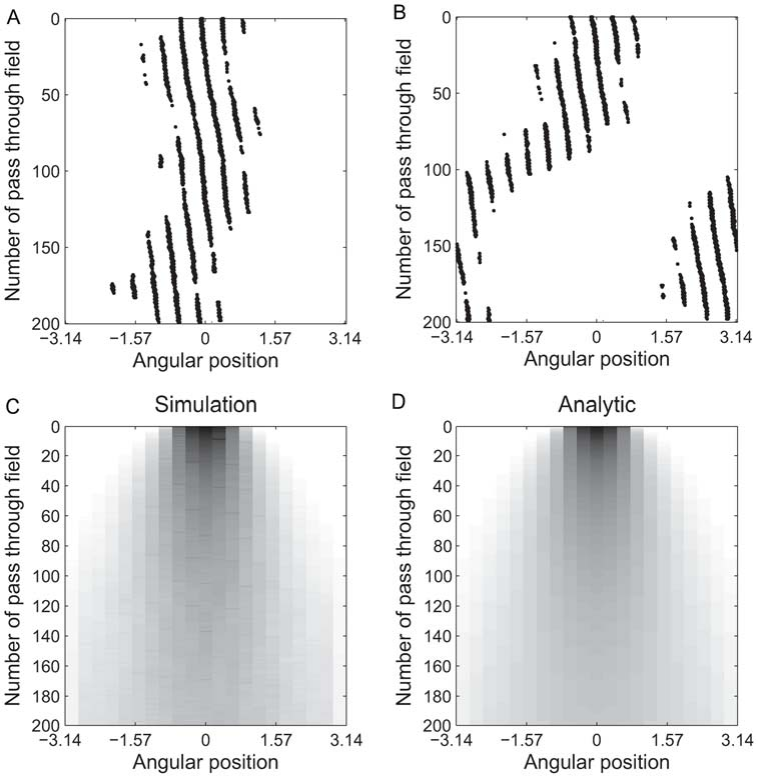
1D simulation results. A, B. Raster plots of two individual runs of the simulation. From top to bottom, each row represents one pass through the grid field. The width of the plot is exactly the distance between the center of two fields so that the path of the virtual rat wraps around each row. Each black dot indicates the position of the virtual rat when the activity of the cell was above threshold during one time step. The positions of the spikes form diagonal lines because the virtual rat moves at a constant rate and the activity of the cell is theta modulated (i.e. the cell can only emit spikes when the baseline oscillation is at its maximum). This effect does not show up in C or D because the starting phase of the oscillations is set randomly for each individual run so that the variations average out. A. A simulation where noise in the system stayed near zero so the field stayed in essentially the same location. B. A simulation where the noise caused the place field to shift almost completely out of phase. C. Average of 3,000 individual, modeled grid cells of the type shown in parts A and B (darker means higher average activity). D. Analytic approximation of the average of many modeled grid cells. Simulations used a fixed 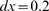. The phase noise variance was arbitrarily set at 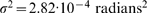 per time step, a level considerably lower than later results in the main text suggest would be present in a biological system.

Here we consider the case of an animal walking in a straight line (Figure 1). We form a raster-like plot where each row, starting from the top and moving downward, shows the activity of the cell on each pass through the field, Figure 2a–b. Variability in the oscillators causes the distance between grid fields to drift over time which manifests as a drift in horizontal position in the plots. In Figure 2a the cumulative noise stays near zero, causing smaller overall changes in the periodicity of the cell than in Figure 2b where the noise has pushed the cell completely out of phase with its correct position by the end of the simulation. The following analysis determines the spatial firing averaged over a large number of such grid cells as noise builds up in the system.

To analytically express the shape of a field, 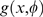, recall that the activity of the grid cell is given by the thresholded sum of two oscillators with phase difference determined by position 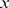 and noise-produced phase shift 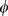. This sum can be thresholded and interpreted as activity in a number of ways. Here we calculate the proportion of time that the sum of the oscillators is above a threshold (i.e. this is a rate-based approximation). Since both cosines have the same frequency, we need only consider one period of the oscillation and can let the frequency equal 1. The noise-free activity at angular position 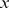 is calculated as
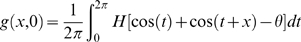
(6)where 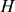 is the Heaviside step function and 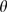 is a firing threshold. The general function is
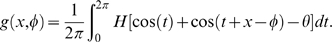
(7)


Notice 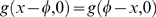 (symmetry) and 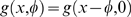 (equivalence of angular positions and phase shifts). The latter means that if the current cumulative noise in a pair of oscillators is, e.g., 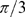 radians, then the spatial firing of the cell would be shifted by the corresponding distance, because angular position directly encodes position between fields.

Given the cumulative noise variance 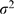 in the system at a given time, we know the probability that the noise will have shifted by 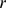 radians is 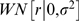. To estimate the population code, we simply sum the shifted spatial firing of a cell by the probability that each specific shift amount has occurred. Specifically, let 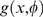 be the activity of a grid cell at angular position 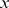 with error in the phase difference of 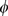 radians (with the field centered at angular position 0). Increasing 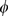 will correspond to shifting the spatial pattern rightward. When there is noise of variance 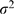, the expected population average activity 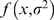 at location 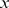 is given by
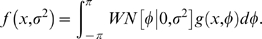
(8)


On the other hand, write can write a population average 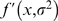 at angular position 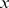 with cumulative variance 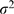, as a convolution of the wrapped normal pdf with 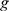: 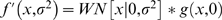 where 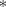 indicates a convolution. Because both 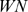 and 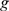 are functions defined on the circle, the convolution needs to integrate only over the interval from 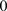 to 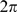:
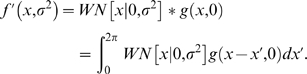
(9)


To see that 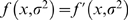, simply recall that 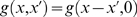. This shows that a convolution (which is efficient to calculate) is equivalent to integrating over the phase differences in the model (which is more time consuming to calculate). However, it will be shown this is only true for the one and two (in 2D) VCO cases.

The convolution approach is compared to the results of simulation of the model in Figure 2c–d. It is clear that the effect of noise on the spatial distribution of the simulated cell's activity is well accounted for by a convolution in this single VCO case.

For each run of the simulation, the virtual rat was restricted to walk at a fixed velocity in a straight line along the preferred direction of the single VCO. Each row in the plot is 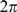 radians wide (the distance between two fields), so that the path of the virtual rat wraps around the figure. The rat was started exactly halfway between two fields (corresponding to the upper left corner of this plot).

Although the cumulative noise variance increases on every time step, it grows sufficiently slowly that we kept the value of the variance constant for each pass through the grid field (each row in Figure 2d). The number of steps from the starting point to the center of the field nearest to the virtual rat was used to calculate the noise variance (number of steps times variance accumulated per step). A noise kernel was calculated from this variance. The interval of phases 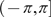 was divided into the same number of bins as was position. The WN pdf was integrated over the range of each such phase bin. This resulted in a vector of probabilities that the current value of the cumulative noise was in each phase bin. For example, had there been four bins and a cumulative noise variance of 1 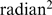, the probability for the first bin would be 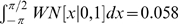. The whole vector of probabilities in this case would be 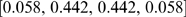. The convolution of this vector with the noise-free spatial distribution would give one row in Figure 2b (though the figure was made using 17 bins, not four).

### Two VCOs

**Figure 3:**
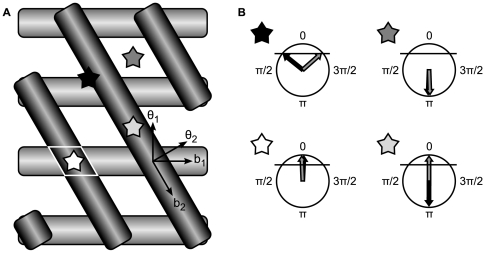
Coding 2D angular position. Positions on a plane are coded using two phase differences (black and gray bands on left, arrows at right). A. The gray VCO's preferred direction is up-down, the black VCO's preferred direction is at an angle of 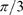 to the gray VCO. A band occurs where the phase difference of one VCO is near 0. A field (white outline at white star) occurs where all the bands intersect (i.e. where all phase differences are near 0). Arrows indicate the preferred directions of the two VCOs (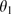 and 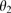) as well as the perpendicular directions along which the bands extend (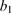 and 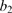). B. The phase differences corresponding to four locations indicated by stars are illustrated. Instead of showing phase of the oscillators, the arrows indicate the phase difference between a VCO and the baseline oscillator. The cell can be thought of as firing when both phase differences are sufficiently near zero (threshold indicated by the horizontal line).

In two dimensions, the activity of a pair of oscillators, which looked like a sequence of fields in 1D, now appears as a sequence of parallel bars or bands. Two sets of bands at an angle of 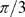 relative to each other are shown in Figure 3. Along a line parallel to a band the phase difference of the oscillator pair does not change. The center line of the bands themselves corresponds to a phase difference of 0. Thus the bands are angled orthogonal to the preferred direction of the corresponding VCO, as it is along the preferred direction that the phase difference changes most quickly. The width of the band as drawn is given by the range of phase differences where the band is over threshold (sufficiently close to 0).

Figure 3 shows the phase differences corresponding to four different locations. The grid cell has a field where bands of both directions overlap (indicated in the figure by a white parallelogram), which is equivalent to saying there is a field where both phase differences are over threshold.

To understand the effects of noise, it is fruitful to consider noise introduced into only one of the two VCOs with no noise in the baseline oscillator. Consider two VCOs with noise in only the VCO encoding distance in the up-down direction (with horizontally-directed bands). This will cause a “blurring” effect on the population level along the direction orthogonal to the preferred direction of that VCO.

**Figure 4:**
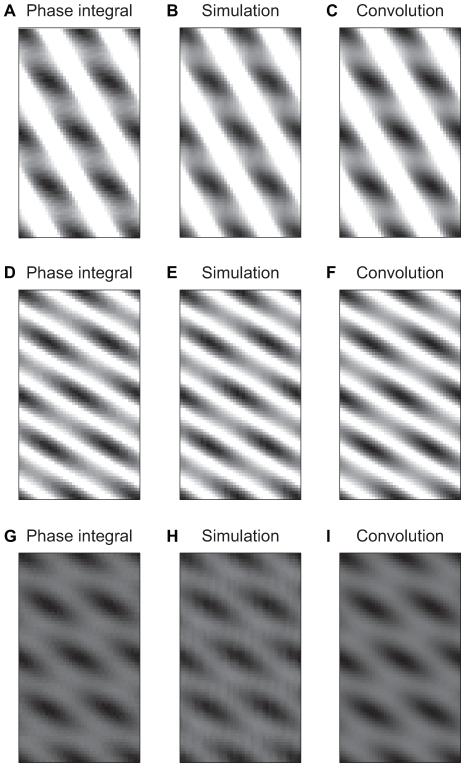
2D population activity. Comparison of population-level activity calculated in three equivalent ways and under different noise conditions. A–C. Population representation of space when noise is introduced only into the VCOs coding the vertical direction. Because the diagonally-directed VCOs are not accumulating noise, the fields stay fixed along the direction perpendicular to the bands. Instead, the blurring of the response occurs along the direction of the bands of the VCOs that do not receive noise. D–F. Population representation when noise is introduced only into the baseline oscillation. G–I. Population representation with noise in the baseline oscillation and the VCOs. A, D, G. Analytic solution using an integral over the phase shifts due to noise. B, E, H. Average population response from running the model 1,000 (B and E) or 5,000 (H) times. C, F, I. Analytic solution using a spatial convolution. Where there is cumulative phase noise in an oscillator it is sampled from a normal distribution with variance 2.5 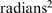.

An average of 1,000 such simulations is shown in Figure 4b. Because there was only noise in the encoding of vertical distance travelled, the diagonal bands do not shift. Since the neuron fires only when both phase differences are near 0, this means that the population code is constrained to still fire along that diagonal band. The population-level representation of space in this case is blurred along the band that does not receive noise. This may be counterintuitive, as one might expect the noise to cause a blurring along the VCO's preferred direction.

To model the effects of noise on the population level, we can directly calculate the average spatial pattern by computing the integral of the activity of the model over the possible phase shifts due to noise (Figure 4a,d). Let 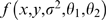 be the average activity of a population of neurons at location 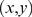, 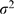 the variance of the cumulative noise, and 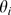 the preferred directions of the two VCOs. Let 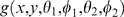 be the analogous activity of a single cell where 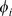 are the current phase difference with respect to baseline of the two VCOs. Then
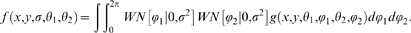
(10)


Or if a noise covariance matrix of 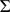 is used to account for noise in the baseline oscillation,
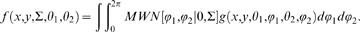
(11)


Consider also the special case of noise only in the baseline oscillation. Then the noise in the two VCOs is always identical and causes an equal spatial shift along both band directions. The blurring thus occurs along a direction halfway between the directions of the two bands.

As in the previous section, we can model this blurring as an equivalent convolution. Each pair of phase differences corresponds to a unique angular position. Shifting one phase difference by a small amount translates the fields by a small amount (but does not change the overall pattern), so integration over the phase differences can be carried out by integrating over shifts of the spatial pattern in an appropriate direction. A minor complexity arises because the spatial shift does not occur in the direction encoded by the phase difference that is being shifted (which was true for the one VCO case). Instead the convolution representing noise along one VCO is directed along the band of the other VCO (and vice versa). To account for noise in the VCOs, one would convolve along the direction of each of the bands in this manner. To account for noise in the baseline oscillator, one would then convolve along the direction halfway between the directions of the bands. See Text S1 in the supplemental material for further technical information regarding the spatial convolution. See Video S1 for an example of the effects of noise on a single grid cell with 2 VCOs (but no baseline noise) in contrast with these population-level effects.

Thus when there are two VCOs encoding a two dimensional position, one can integrate over a range of phases by convolving over the corresponding spatial shifts, as demonstrated in Figure 4c,e. This is no longer true when three or more VCOs are used to encode a two dimensional position. As is shown in the next section, the spatial pattern changes in a non-translational way as the phases of the VCOs are changed (see Videos S2 , S3 , S4, S5), so simply summing over translated spatial patterns cannot capture the results.

We can finish by comparing the simulation results with both the convolution approach and the double integral over phase shifts. Figure 4 shows that both analytic results agree and match the simulation results.

### Three or More VCOs

Even though two VCOs are sufficient to encode position in a two-dimensional space, use of only two VCOs causes diamond-shaped fields which are not seen in experimental data. Using three VCOs can produce nearly circular shaped fields, but there is no principled reason that a cell should have only three VCOs so we simulate conditions of up to 93 VCOs in one cell. It is unclear how many VCOs a real grid cell would have and the number may depend on the specific biological oscillator creating the grid pattern. For instance, layer II stellate cells have 4–8 thick, proximal dendrites [35]. However, as each of these dendrites ramifies many times, it is not clear whether a single dendrite may contain, e.g., multiple redundant oscillators with the same preferred direction.

When a neuron has three or more VCOs, noise has three main effects on the spatial firing of the model. First, noise can cause a drift in the spatial phase of a grid as in the previous sections. As will be shown, it can also cause new spatial patterns to form (such as new fields appearing in between existing fields) and can cause fields to deform, shrink, or vanish completely. These three effects can be seen clearly in Videos S2–S5, which show single cell examples with 3, 12, 36, and 72 noisy VCOs, respectively.

We examine these effects using three approaches. First we use a measure that captures all of the above effects to broadly summarize the firing of the model. We also look at the proportion of cells that stop firing entirely. Finally, we analyze the drift in spatial phase independent of the other effects.

We start with an imperfect but informative summary measure of the spatial firing changes of a cell: the mean distance between each spike and the center of where the nearest field would be in a noiseless condition. We calculate analytically where the centers of the fields should be (the lattice points of unit vectors in the VCOs' preferred directions) and then calculate the mean distance of each spike from whichever of those centers is nearest. This measure increases as fields increase in size, appear in new locations, or as the fields move away from their correct positions. The measure decreases as fields decrease in size and equals 0 when there are no spikes. The measure is imperfect because it does not distinguish among these effects and because, e.g., there are conditions under which smaller fields can produce a higher value. Nevertheless it is useful as a broad summary of the activity of cells.

**Figure 5:**
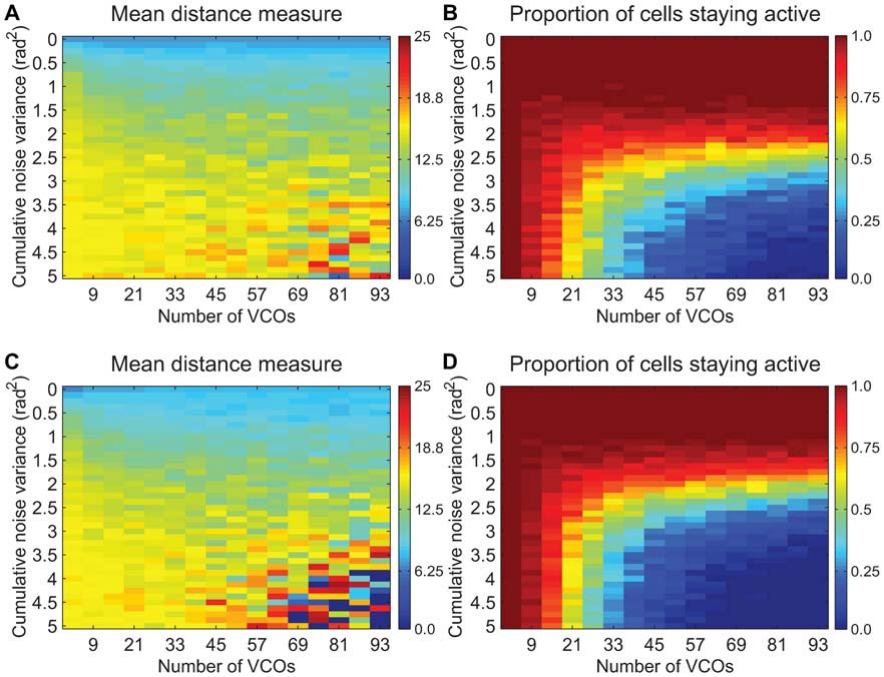
Simulations as number of VCOs and amount of noise are varied. In each plot, the x-axis indicates the number of VCOs used in a simulation (from 3 to 93 in increments of 6) and the y-axis indicates the variance of the noise in 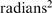. A, C. The average (over multiple runs) of the mean distance of each spike to the center of where the nearest field would be if there were no noise is plotted (redder means higher mean distance; darkest blue indicates points where all cells stopped firing so the measure equaled 0). B, D. Plots show the proportion of cells that remain firing despite the noise (bluer means higher proportion of cells stop firing). A, B. Sum activation rule (using a threshold proportional to the number of VCOs). C, D. Rectified-product activation rule. Notice the general pattern of results is independent of the activation rule (at least for these two rules). Plots of the same type have the same color scale. Each point is the average of 150 simulations with the corresponding parameters.

**Figure 6:**
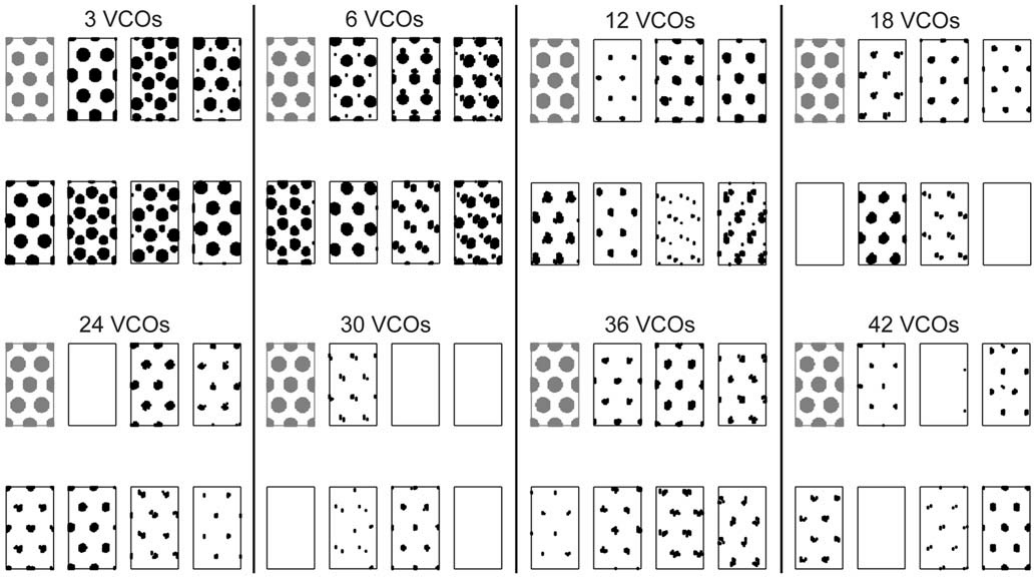
Example spatial firing as number of VCOs is varied. Two-dimensional spatial firing plots of seven example simulated cells with varying numbers of VCOs as the amount of noise is held fixed at a variance of 2.5 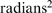. Black indicates a location where a cell is active. The plots correspond to sampling along one row of Figure 5. These do not show cumulative spikes, but rather the spatial firing as it would appear starting with phase errors distributed with this level of variance, but then accumulating no more noise. When there is no noise (shown in gray for each set), all conditions have essentially the same spatial pattern.

This measure is plotted over a range of values of noise variance and number of VCOs in Figure 5. The measure was only averaged over runs where there was at least one spike. In row zero there is no noise and the measure is lower in this row than almost any other location in the plot. Generally the mean distance increases as the noise variance increases, except where the majority of the cells stop firing. In those cases (lower-right corner of mean distance measure plots), there are so few samples to average over that the value is sometimes very high and sometimes very low. Especially for low amounts of variance, there is also a trend toward lower mean distance measures as the number of VCOs increases, so greater numbers of oscillators seem to decrease the effects of noise (see diffusion analysis later). This is particularly clear in Figure 6, showing examples of the spatial activity as the number of VCOs varies while the noise level held fixed.

To see why large amounts of noise cause cells with a large number of VCOs to stop firing, consider that the activation rule of the model is approximately: the cell fires when a sufficient number of the phase differences are nearly zero. Notice that the shutting-off point in Figure 5 is independent of the number of VCOs (except for small numbers of VCOs). It is a function only of the amount of noise and occurs as the distribution of shifts in phase difference due to noise becomes less sharply peaked and more uniformly distributed. As this occurs it becomes increasingly unlikely that there will be any location where enough of the phase differences are near zero at the same time.

However, up until and even after the point where the phase differences are sufficiently scattered that the cell stops firing, the mean of the set of phase differences with a common preferred direction tends to stay near the correct position. Each oscillator is an independent, noisy estimate of the true position. This suggests that increasing the number of oscillators increases resilience to noise (up to the point where the cell stops firing completely).

We can examine this more closely by following [7] in examining the diffusive character of the effects of noise. In Text S2 in the supplemental material we derive analytically that the expected square of the distance that the encoded spatial phase drifts equals 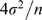 where 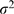 is the variance of the cumulative noise and 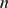 is the number of VCOs, when the preferred directions of the VCOs are all at 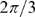 increments. This arrangement of preferred directions causes the baseline noise effects on drift to cancel out. For other sets of preferred directions, diffusion due to baseline noise can greatly overwhelm diffusion due to noise in the VCOs (see Text S2). As the variance is expected to increase linearly with time (per our assumption of independent noise on each step), the distance the encoded spatial phase drifts over a time interval will be proportional to the length of that interval, making the noise diffusive just as in the continuous attractor models. The analytic solution also directly shows that the diffusion due to noise decreases as the inverse of the number of VCOs, confirming the intuitive usefulness of having multiple independent estimates.

Earlier we claimed that the average spatial pattern over a population cannot be calculated with a convolution when there are more than two VCOs encoding a two dimensional position. However, the phase integral approach can still be used to calculate the average population activity. Let 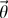 be a 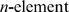 vector of the preferred directions of each oscillator and 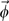 an 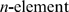 vector of phase difference shifts for each oscillator (where 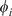 is the 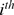 element). Then compute the 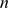 nested integrals, running over each possible phase vector 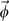.
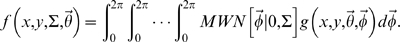
(12)


Notice that for large numbers of VCOs, numerical calculation of this multiple integral is computationally expensive (calculating 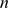 VCOs discretized into 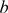 phase bins requires 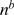 calculations of 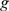).

### Comparison to Experimental Data

**Figure 7:**
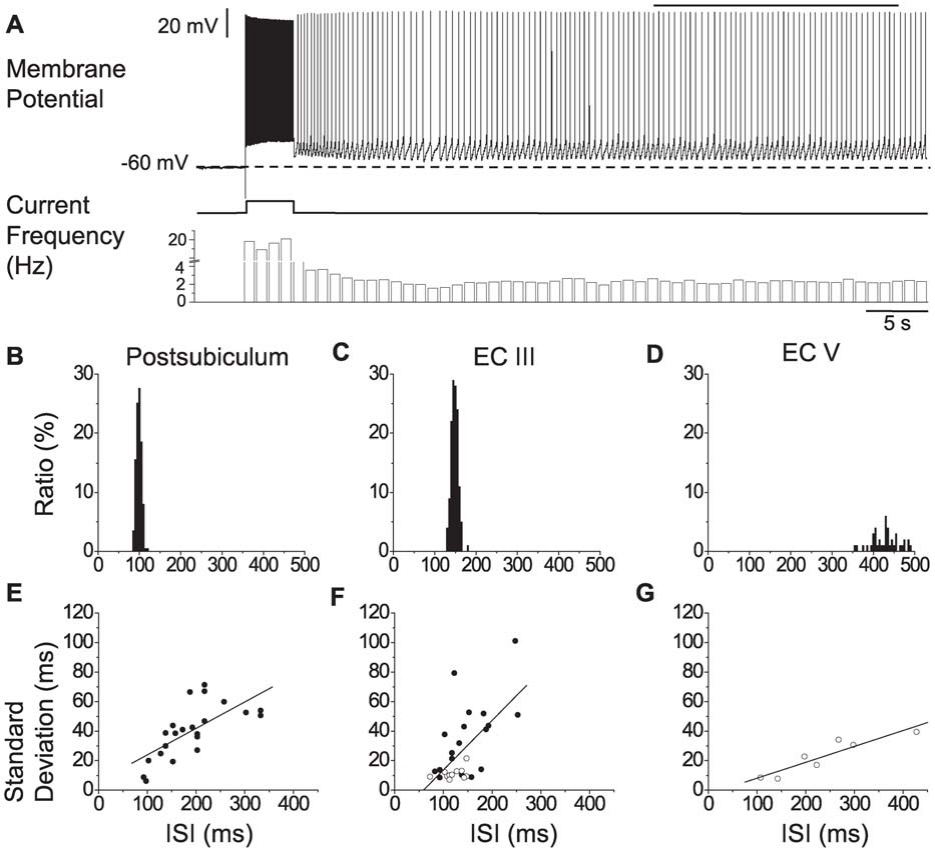
Summary of experimental data. Regularity of inter-spike interval (ISI) of persistent firing. A. Membrane potential trace shows an example of persistent firing recorded from entorhinal cortex layer V. The straight line on top of the membrane potential plot shows the 20 s section from which ISIs were measured. The current trace shows the brief current injection (2 s, 200 pA) which triggered persistent firing. The frequency plot shows frequency of firing in 1 s bins. B–D. Example ISI histograms measured from 20 s section in cells in the: B. postsubiculum, C. entorhinal cortex layer III (ECIII), and D. entorhinal cortex layer V (ECV). For each group, the cell with the highest estimated stability time (not necessarily the lowest standard deviation) was chosen. E–G. Linear fit to standard deviation of ISI as the function of the peak ISI for: E. postsubiculum (t-test for correlations; 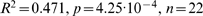), F. ECIII (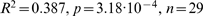), and G. ECV (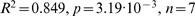). Filled circles: Cells from whole-cell patch recording. Open circles: Cells from sharp-electrode recording.

So far we have examined noise measured in radians, but have not related these noise values to biologically relevant units. We now examine variability in the interspike interval (ISI) in persistent spiking neurons in postsubiculum as well as in entorhinal cortex layers III and V, shown in Figure 7. We also examine noise in terms of the period of subthreshold membrane potential oscillations using the experimental data from [20] and examine theta frequency bursting of medial septal neurons using data from [36].

As Figure 5 showed, independent of the activation rule or number of VCOs, there is a threshold level of cumulative noise variance in the range of roughly 2.5–3.5 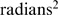 past which a grid cell is no longer expected to be accurately encoding position. We take 2.5 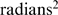 as a threshold level of noise variance. Although the value may be seem arbitrary, it corresponds to a standard deviation of nearly 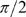, and at that point there is less than a 50% chance that the phase difference encoded by a pair of oscillators is within 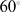 of the correct value (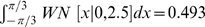), suggesting it is a very reasonable value to use. Because there is a relationship between the number of VCOs and the drift due to noise, however, this threshold value is only an approximation. Strictly, a grid cell model with only a few VCOs would have a lower noise threshold because of the resulting greater sensitivity to noise.

Next, [2] reported that grid cells can maintain fairly accurate encoding of position for at least 10 minutes in darkness (although input from other sensory modalities may have contributed to the stability of the firing). A more liberal benchmark is that the phase difference encoded by oscillators should only last 60 seconds [21], although it is unclear exactly how this value was determined. Alternative constraints come from the head direction and place cell systems, which are interconnected with the grid cell system [24],[37]. Head-direction neurons in the lateral dorsal nucleus of the thalamus maintain their preferred directions in darkness for as little as 2–3 minutes [38]. Head-direction neurons in the anterior thalamic nucleus have comparable stability times [39], though they were reported to vary considerably depending on behavior and sensory input. Place fields from dorsal hippocampus rotated around a circular arena in the dark on the same time scale ([39]; likely a direct reflection of the drifting head direction signal). On the other hand, another study [40] found that most hippocampal place cells (24 of 28) were stable in darkness for at least 8 minutes, though non-visual cues may have been present. Interpreting these results can be difficult because generally no quantitative attempt is made to characterize the drift (with at least one exception [41]).

Using these as rough guidelines we can estimate an upper limit to the rate at which noise can accumulate in the model. The cumulative variance must not exceed 2.5 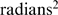 after 2 minutes (120 seconds) of behavior, which corresponds to 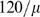 periods of the oscillators with mean period 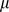. Then the maximum variance introduced on each oscillation must be less than 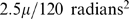. Note in particular that this value is linear in both the maximum variance as well as in the duration of stability.

Consider a pair of oscillators with a fixed baseline oscillation of period 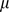 seconds and an imperfect VCO where the period of the VCO is normally distributed with mean period 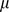 seconds and variance 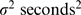. For now we assume that the baseline oscillator is perfect (zero variance). If the two oscillators begin in phase with each other and receive no velocity input, the difference between the time of the 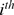 peak of the baseline oscillation and the 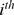 period of the second oscillation will be the sum of the differences in period of each of the preceding 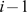 oscillations. Each of these temporal differences are distributed as 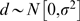. We can convert the temporal differences to phase differences by dividing by the baseline period 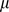 seconds and multiplying by 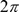 radians for a distribution of 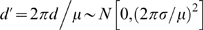. Instead, if both oscillators are noisy with the same mean period 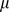 and variances 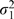 and 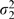, then the difference of the lengths of the periods is distributed as the difference between two normally distributed variables, which is again normally distributed with mean zero and variance 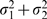. The corresponding phase differences are then distributed as 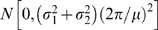 or simply 
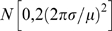
 if the variances are equal. Inserting experimentally measured 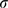 and 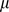 (in seconds), we can test whether 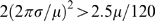, in which case the system is expected to be too noisy to maintain a stable spatial pattern over the specified amount of time (e.g. 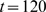 seconds here).

The inequality can be rearranged to 
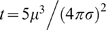
 seconds. This gives the number of seconds until the variance reaches the noise threshold of 2.5 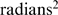. In Table 1, we calculate this amount of time 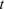 for a number of biological oscillators. It is worth noting that one oscillatory interference model [13] created a grid pattern without the use of a baseline oscillation (but see [42]). In this case, the cumulative variance would be halved, so the resulting estimated stability times would be 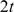 (i.e. double the values given in Table 1).

**Table 1:** Estimated stability of grid cells for various biological oscillators.

Oscillator; recording method	Case	Mean (s)	Std. (s)	Stability time (s)
ECII smpo; whole-cell patch	dorsal average ( 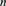 = 18)	0.126	0.051	0.024
	ventral average ( 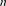 = 17)	0.151	0.074	0.020
	most stable cell	0.149	0.055	0.035
	median cell	0.177	0.086	0.027
	least stable cell	0.194	0.123	0.0153
ECIII ps; sharp electrode	average ( 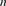 = 11)	0.121	0.0113	0.464
	most stable cell	0.143	0.009	1.14
	median cell	0.113	0.01	0.457
	least stable cell	0.0725	0.009	0.152
ECIII ps; whole-cell patch	average ( 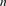 = 18)	0.149	0.036	0.081
	most stable cell	0.158	0.009	1.54
	median cell	0.118	0.021	0.118
	least stable cell	0.123	0.079	0.009
ECV ps; sharp electrode	average ( 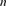 = 7)	0.125	0.089	0.008
	most stable cell	0.428	0.040	1.55
	median cell	0.298	0.031	0.872
	least stable cell	0.198	0.023	0.465
MS 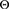 burst [36]; single electrode	average ( 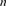 = 12)	0.132	0.028	0.093
POS ps; whole-cell patch	average ( 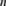 = 22)	0.238	0.023	0.807
	most stable cell	0.0975	0.006	0.828
	median cell	0.218	0.047	0.149
	least stable cell	0.188	0.067	0.047

Means and standard deviations of oscillator periods taken from the literature are averages over multiple cells (average of the mean period lengths and average of the standard deviations). Means and standard deviations taken from our original experimental data are given as both the average mean and average standard deviation over all cells as well as the single cells with the longest, median (where there were an even number of cells, the greater of the two “middle” cells is used), and shortest estimated duration of stability. Expected stability time is calculated using the equation 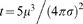 as derived in the text for noise in pairs of oscillators (stability times should be doubled in models with no baseline oscillation). Experimental data is from this manuscript except where a reference is provided. For comparison, head direction cells are stable for around 120 seconds in the dark [38],[39], two orders of magnitude longer than the expected stability of the best of these biological oscillators. Abbreviations: standard deviation (Std.), entorhinal cortex layer II, III, or V (ECII, ECIII, ECV), postsubiculum (POS), medial septum theta bursting cell (MS 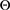 burst), subthreshold membrane potential oscillations (smpo), persistent spiking (ps).

Consider the calculation for persistent spiking, an example of which is shown in Figure 7a. In this study persistent firing was induced with a brief supra-threshold current stimulation. ISIs were measured in a 20 s window starting 7–30 s after current injection offset (indicated by a line over the trace in Figure 7a) to avoid the initial drift in frequency that occurs after the stimulation [43]. The recordings were performed in the presence of synaptic blockers so the measured stability is interpreted as indicating the ability of the single neuron to maintain a stable firing frequency as if the animal were maintaining a constant velocity. Figures 7b–d show ISI histograms from the most stable cell in each of the three regions (note that the most stable cell is not necessarily the one with the lowest ISI standard deviation). As shown in Figures 7e–g, the ISI standard deviation increases linearly with the mean ISI. The most stable cell had a mean ISI of 0.428 s and a standard deviation of 0.040 s. Substituting in, 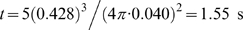. Two of these cells acting as an oscillator pair in the model would be expected to reach our threshold level of cumulative noise in 1.55 seconds, about 3.6 cycles.

To see why this is the case, consider that the standard deviation of 0.040 s is 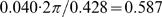, corresponding to a variance of 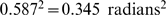 in one oscillator. Because there is noise in both oscillators, the distribution of the phase difference noise has twice the variance of one of the oscillators, so the phase difference distribution accumulates a variance of 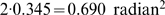 during each cycle. The threshold level of variance is 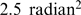, so the phase difference will reach this level in 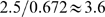 cycles. For comparison, in order for the expected stability time to equal 120 s, the standard deviation would need to be 0.0045 s.

Previous work [20] examined the peak-to-peak time intervals describing the period of the subthreshold membrane potential oscillations in medial entorhinal cortical later II stellate cells (putative grid cells). Figure 5E in that paper shows that the standard deviation varies linearly with the mean oscillation period (although it is not clear that the periods are actually normally distributed as they seem to have a positive skew, see Discussion). The rate of accumulation of noise would be expected to differ along the dorsoventral extent because the slope of that line does not equal 1. The slowest rate of accumulation (lowest variance) is in the dorsalmost area. In Table 1 we give stability values for the longest and shortest stability times, as well as values using the average dorsal and ventral values given in [20].

Another oscillatory interference model [19] suggests the oscillators may lie in subcortical regions known to be generators of the theta rhythm, such as the medial septum, reticular formation, etc. [36] report values on variability of theta burst firing of medial septal neurons. The variance was smaller in their alcohol-non-preferring rats, where the mean inter-burst interval was 0.0824 s with a standard error of 0.0054 s (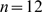) giving a standard deviation of 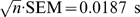. The mean burst duration was 0.0496 s with a standard error of 0.0061 s giving a standard deviation of 0.0211 s. We combine the reported length of the bursts and the interval between bursts to find the mean period and variance of the oscillator as a whole. Thus the mean oscillator duration was 0.132 s and its standard deviation 0.028 s, which results in a very low estimated stability time. However, some of this variance could arise from variations in the animals' running speeds (for instance, see Figure 2 in [44]) as it is not clear whether [36] controlled running speed in all or only in some of their data. In the oscillatory interference model, this velocity-related variance is not noise, but instead reflects the function of the model.

The ages of the animals used in our experimental recordings are young enough that developmental processes might still be occurring, possibly affecting oscillatory variability. Although the ages of the animals used in our sharp electrode recordings are only known approximately, the exact age of each animal in the whole-cell patch condition was known. Figure S1 plots mean and standard deviation of period length as well as estimated stability times versus age for each cell. There was no statistically significant correlation between age and any of these three values.

## Discussion

We have examined the effects of noise on the oscillatory interference type models of grid cell activity. When one VCO is encoding position along one dimension, or two VCOs in two dimensions, noise produces a convolution-type blurring on the population level. When more than two VCOs are used to encode two-dimensional positions, the convolution approach fails to match the simulations and the expected population representation must be integrated over the possible phase shifts due to noise. In these cases noise causes a number of effects on spatial firing, most prominently the appearance of new spatial fields (Figure 6). Another clear effect is that cells with medium and large numbers of VCOs stop firing as noise accumulates. We saw that when multiple VCOs have the same preferred direction, they work as independent estimators of the true angular position. This results in a more reliable spatial signal both when individual cells have more VCOs and when networks have more grid cells with identical parameters. This is a strength of oscillatory interference models over continuous attractor models, in which errors affect all cells in the same way [7]. This redundancy also addresses the question asked in [21] as to why oscillatory interference models would need more than a handful of neurons.

However, our analysis of the variability of biological oscillators presents a challenge to current oscillatory interference models. As informally considered in [21] for the case of subthreshold membrane potential oscillations, the amount of variability in the neural systems that we have examined is too high to maintain stable grid firing on the time scales seen in animals in the dark. It is not presently clear to what extent resetting of the grid network by external cues [11],[22],[23] is responsible for the longer stability times seen *in vivo* or to what extent resetting could extend the low stability times we have estimated in this manuscript.

A general mechanism for hippocampal or sensory-driven resetting forms associations (possibly bidirectionally) between patterns of hippocampal (or sensory) activity occurring at each location the animal has visited with the co-active pattern of grid cells [22],[23],[45]. The system can then correct noise-driven drift in the grid system during navigation in a familiar environment by biasing activity in the overall place system to reflect sensory input. In principle the system could be biased in the other direction, so a given grid cell activity pattern could activate the associated hippocampal or sensory representations, resulting in a form of spatial memory. In a novel environment, there would be no familiar cues to reset the system so there would be no resetting of the system for some initial period (likewise in, e.g., darkness or perhaps even general locomotion while attention is not focused on navigation). It is during this time when the grid system is presumably performing proper path integration that the variability of the VCOs has the greatest effect. In the oscillatory interference model, a simple mechanism would reset each grid cell when the animal moves near enough to the center of a grid field [11], so each grid cell would be reset separately, and the noise in the VCOs would represent the amount of noise accrued since the animal last passed near the center of one of a grid cell's fields. A resulting prediction is that when an animal is stationary for a period of time, noise may cause the fields of neighboring, un-reset grid cells to drift past the animal's current position. That is, grid cells may appear to begin firing spontaneously and randomly when the animal is motionless. Similarly, in an environment sufficiently small that some cells do not have a field in the environment, those cells should also show a constant random drift.

Future experimental work should examine the stability in cue-controlled circumstances to more precisely fix the limits of temporal stability. Additionally, experimental work can easily examine the variance and mean period of biological oscillators that are proposed as a substrate for oscillatory interference models, especially in subcortical regions associated with the control and expression of the theta rhythm, such as the reticular formation. Autocorrelation histogram damping time constants for many regions which show oscillations in the theta band have been reported [46]. The damping time constant of an oscillator's autocorrelogram is directly related to its phase noise variance so these numbers can provide a rough measure of the oscillations that might be found in those regions (e.g. see [47],[48] for useful mathematical results). Neurons of the medial septum-diagonal band of Broca had the longest damping time constant (0.34 s), suggesting they were the least variable in their theta-modulated firing [46]. Other regions and their time constants given in [46] were antero-ventral nuclei of the thalamus (0.29 s), CA3 (0.28 s), fascia dentata (0.27 s), presubiculum (0.19 s), posterior limbic/retrosplenial cortex (0.12 s), and anterior limbic cortex (0.09 s). These are quite low values, but because they are averaged over many cells the variability of the best units might be much lower (notice the difference in orders of magnitude between individual best cells and population averages in Table 1).

One issue raised in the analysis of *in vivo* oscillators is that their variability might reflect multiple noise sources, some of which are correlated among multiple oscillators and some of which are uncorrelated (e.g. intrinsic to the cell). The distinction is relevant because changes in frequency that are identical in two oscillators will not affect the difference in their phases. It is the uncorrelated noise component that introduces the type of phase difference errors between oscillators we examined in this manuscript. Recording from a single oscillator *in vivo* does not provide enough information to distinguish the two, so the total variance of oscillations recorded *in vivo* may appear larger than the component of the variance that affects stability in the manner we have analyzed (i.e. the oscillator may appear to be much less stable than it intrinsically is). The effects of correlated noise on the model and the magnitude of the noise *in vivo* should be examined in the future, as it is known that correlated noise between similar oscillators can produce a synchronizing effect (stochastic synchrony; e.g. [32],[49],[50]), which may produce further phase difference errors.

One possible direction for theoretical work would be forming hybrid networks that combine the advantages of oscillatory interference models with those of continuous attractor models. It is also possible that circuit level interactions can decrease variance by creating an oscillatory circuit that pools either synchronized/coupled or independently-noisy oscillators with the same period but possibly different phases. For instance, our own unpublished simulations show that synaptically-coupled persistent spiking neurons can synchronize their spiking such that the periods of the population as a whole have a lower variance than the periods of individual, uncoupled neurons and the variance decreases as the size of the populations increase. In any case, if such mechanisms can reduce variability, the reduced-variance oscillation should be experimentally detectable, and the approximations derived earlier may be useful to evaluate candidate circuit level mechanisms whose oscillatory periods can be easily measured but where it may be technically difficult to carry out a full-scale simulation of them to produce grid cells. However, given that the study of grid cells is still in its infancy, it is not unlikely that current models are merely rough starting points for the understanding of a system whose full complexity may not yet be revealed.

At present, the published reports of grid cells describe recordings from adult rats (e.g. 3–5 months old; [3]), but our slice recordings were performed in rats as young as postnatal day P17. These animals were used because younger animals (e.g. less than 30 days old) produce higher quality brain slices [51]. Preliminary data [52] suggests that the first appearance of grid cells is around P17, but we are not aware of estimates of stability in those grid cells. Oscillatory interference models, based on our present data, would predict these cells would have spatial activity stable only for a few seconds. There is a possibility that developmental processes continuing past this age might cause oscillatory variability to change in older animals. However, the development of graded persistent firing in entorhinal cortex layer V has been examined [53] and in many respects the cells were leveling off by around P16. This nearly the same point in development where subthreshold membrane potential oscillations start appearing (P14–P18, [54]), which change little beyond P28. It is interesting to note that [54] suggested the development of these oscillations may be driven by an increased number of persistent sodium channels. As the variance of the channel noise current is proportional to the number of channels [29], the magnitude of the channel noise may actually be increasing during development (although the ratio of the standard deviation to the mean would be decreasing). While this is not conclusive evidence it does, combined with our lack of correlations with age, support the claim that the cells we examined were mature enough to show the same relevant phenomena as adult neurons.

In our analysis of biological oscillators, we assumed that the noise took the form of normally distributed oscillator periods, however the period histograms in [20] as well as some of our own persistent spiking ISI histograms had a shape that appears positively skewed (longer right tail). This shape is consistent with an oscillator in which the frequency of each period and not its wavelength (duration of each period) is normally distributed, which would make the wavelength distributed as the inverse of a normally distributed variable. The skew in the period distribution would cause the average wavelength to be longer than the wavelength of the mean frequency. This would mean the error terms would have a nonzero mean, which would cause a constant shift in the encoded phase differences. Simulations in [20] examined noise with a non-zero mean in an oscillatory interference model. There the noise was shown to quickly destroy any spatial firing pattern because of this constant drift. This sensitivity to noise with a non-zero mean is not a failing of this particular model as it is reasonable that any path integration model should fail if there is an uncorrected directional bias (non-zero mean) in the noise in its velocity input or position encoding. This non-normal distribution of oscillation wavelengths is not accounted for by our analysis and may cause an even larger decrease in stability than the apparent increase in variance due to the long right tail.

It is not yet clear what mechanisms underlie the spatial firing patterns of grid cells. However, we have shown the common criticism [7],[13],[20],[21] that oscillatory interference models are particularly sensitive to noise is not true for sufficiently small levels of noise when there are many redundant VCOs, but experimentally determined levels of noise are significantly higher and suggest modified oscillatory interference models are needed to account for the observed stability of grid cells. Nevertheless, there are still many open questions regarding the basis of the hexagonal grid firing pattern and only future experimentation can settle these issues.

## Supporting Information

Text S1 Technical notes on the 2 VCO convolution
(0.06 MB PDF)

Text S2 Diffusion analysis
(0.09 MB PDF)

Figure S1 Scatter plots of experimental data for three different regions of the brain. Estimated stability times are calculated from the oscillator period mean and standard deviation using the equation derived in the text for a pair of noisy oscillators. In the postsubiculum recordings, the correlation r between age and period length (mean ISI) was not significantly different from 0 (Student's t test for correlations, r = 0.005; N = 22; 2-tailed p = 0.98) nor was the correlation between age and period standard deviation (r = 0.056; N = 22; 2-tailed p = 0.79). The entorhinal cortex layer II subthreshold membrane potential oscillation data showed the same results: the correlation between age and mean period length (mean peak-to-peak time) was not significantly different from 0 (r = 0.25; N = 25; 2-tailed p = 0.14) nor was the correlation between age and period standard deviation (r = 0.22; N = 25; 2-tailed p = 0.21). In the whole-cell entorhinal cortex layer III recordings, the correlation between age and mean period was not significantly different from 0 (r = −0.39; N = 18; 2-tailed p = 0.11) nor was the correlation between age and period standard deviation (r = −0.35; N = 18; 2-tailed p = 0.15).
(0.88 MB EPS)

Video S1 Spatial activity of the model with 2 VCOs as noise accumulates. The left side of the video is a spatial firing rate map calculated as described in the text. The darkest blue color means no firing, and darkest red corresponds to the highest firing rate. The text over this plot indicates the variance of the cumulative noise as it builds up from 0 to 3 radians^2^. The right side of the video shows the same region of space but shows the overlapping activity of each VCO (colored red and blue) such that the cell fires when both bands overlap and produce a magenta color (compare to Figure 3 in the text). The changing spatial pattern on the left is a direct result of the noise-induced movements of the individual VCO bands shown on the right.
(2.65 MB AVI)

Video S2 Spatial activity of the model with 3 VCOs as noise accumulates. The left side of the video is a spatial firing rate map calculated as described in the text. The darkest blue color means no firing, and darkest red corresponds to the highest firing rate. The text over this plot indicates the variance of the cumulative noise as it builds up from 0 to 3 radians^2^. The right side of the video shows the same region of space but shows the overlapping activity of each VCO (colored red, green, and blue) such that the cell fires when all three bands overlap and produce a white color (compare to Figure 3 in the text). The changing spatial pattern on the left is a direct result of the noise-induced movements of the individual VCO bands shown on the right.
(2.77 MB AVI)

Video S3 Spatial activity of the model with 12 VCOs as noise accumulates. The left side of the video is a spatial firing rate map calculated as described in the text. The darkest blue color means no firing, and darkest red corresponds to the highest firing rate. The text over this plot indicates the variance of the cumulative noise as it builds up from 0 to 3 radians^2^. The right side of the video shows the same region of space but shows the overlapping activity of each VCO (colored red, green, and blue; the four VCOs of the same color are each drawn at 25% transparency) such that the cell fires when enough bands overlap and produce a white color (compare to Figure 3 in the text). The changing spatial pattern on the left is a direct result of the noise-induced movements of the individual VCO bands shown on the right.
(2.66 MB AVI)

Video S4 Spatial activity of the model with 36 VCOs as noise accumulates. The left side of the video is a spatial firing rate map calculated as described in the text. The darkest blue color means no firing, and darkest red corresponds to the highest firing rate. The text over this plot indicates the variance of the cumulative noise as it builds up from 0 to 3 radians^2^. The right side of the video shows the same region of space but shows the overlapping activity of each VCO (colored red, green, and blue; the 12 VCOs of the same color are each drawn at 1/12 transparency) such that the cell fires when enough bands overlap and produce a white color (compare to Figure 3 in the text). The changing spatial pattern on the left is a direct result of the noise-induced movements of the individual VCO bands shown on the right.
(2.36 MB AVI)

Video S5 Spatial activity of the model with 72 VCOs as noise accumulates. The left side of the video is a spatial firing rate map calculated as described in the text. The darkest blue color means no firing, and darkest red corresponds to the highest firing rate. The text over this plot indicates the variance of the cumulative noise as it builds up from 0 to 3 radians^2^. The right side of the video shows the same region of space but shows the overlapping activity of each VCO (colored red, green, and blue; the 24 VCOs of the same color are each drawn at 1/24 transparency) such that the cell fires when enough bands overlap and produce a white color (compare to Figure 3 in the text). The changing spatial pattern on the left is a direct result of the noise-induced movements of the individual VCO bands shown on the right.
(2.34 MB AVI)

## References

[1] Fyhn M, Molden S, Witter MP, Moser EI, Moser MB (2004). Spatial representation in the entorhinal cortex.. Science.

[2] Hafting T, Fyhn M, Molden S, Moser MB, Moser EI (2005). Microstructure of a spatial map in the entorhinal cortex.. Nature.

[3] Sargolini F, Fyhn M, Hafting T, McNaughton BL, Witter MP (2006). Conjunctive representation of position, direction, and velocity in entorhinal cortex.. Science.

[4] Fuhs MC, Touretzky DS (2006). A spin glass model of path integration in rat medial entorhinal cortex.. J Neurosci.

[5] McNaughton BL, Battaglia FP, Jensen O, Moser EI, Moser MB (2006). Path integration and the neural basis of the ‘cognitive map’.. Nat Rev Neurosci.

[6] Guanella A, Kiper D, Verschure P (2007). A model of grid cells based on a twisted torus topology.. Int J Neural Syst.

[7] Burak Y, Fiete IR (2009). Accurate path integration in continuous attractor network models of grid cells.. PLoS Computational Biology.

[8] Gaussier P, Banquet JP, Sargolini F, Giovannangeli C, Save E (2007). A model of grid cells involving extra hippocampal path integration, and the hippocampal loop.. J Integrated Neurosci.

[9] O'Keefe J, Burgess N (2005). Dual phase and rate coding in hippocampal place cells: Theoretical significance and relationship to entorhinal grid cells.. Hippocampus.

[10] Blair HT, Welday AW, Zhang K (2007). Scale-invariant memory representations emerge from moiré interference between grid fields that produce theta oscillations: A computational model.. J Neurosci.

[11] Burgess N, Barry C, O'Keefe J (2007). An oscillatory interference model of grid cell firing.. Hippocampus.

[12] Hasselmo ME, Giocomo LM, Zilli EA (2007). Grid cell firing may arise from interference of theta frequency membrane potential oscillations in single neurons.. Hippocampus.

[13] Hasselmo ME (2008). Grid cell mechanisms and function: Contributions of entorhinal persistent spiking and phase resetting.. Hippocampus.

[14] Giocomo LM, Zilli EA, Fransén E, Hasselmo ME (2007). Temporal frequency of subthreshold oscillations scales with entorhinal grid cell field spacing.. Science.

[15] Klink R, Alonso A (1997). Muscarinic modulation of the oscillatory and repetitive firing properties of entorhinal cortex layer II neurons.. J Neurophysiol.

[16] Tahvildari B, Fransén E, Alonso AA, Hasselmo ME (2007). Switching between “On” and “Off” states of persistent activity in lateral entorhinal layer III neurons.. Hippocampus.

[17] Yoshida M, Fransén E, Hasselmo ME (2008). mGluR-dependent persistent firing in entorhinal cortex layer III neurons.. Eur J Neurosci.

[18] Yoshida M, Hasselmo ME (2009). Persistent firing supported by an intrinsic cellular mechanism in a component of the head direction system.. J Neurosci.

[19] Blair HT, Kishan G, Zhang K (2008). Conversion of a phase- to rate-coded position signal by a three-stage model of theta cells, grid cells, and place cells.. Hippocampus.

[20] Giocomo LM, Hasselmo ME (2008). Computation by oscillations: Implications of experimental data for theoretical models of grid cells.. Hippocampus.

[21] Welinder PE, Burak Y, Fiete IR (2008). Grid cells: The position code, neural network models of activity, and the problem of learning.. Hippocampus.

[22] Redish AD, Touretzky DS (1997). Cognitive maps beyond the hippocampus.. Hippocampus.

[23] Redish AD (1999). Beyond the cognitive map: From place cells to episodic memory..

[24] van Strien NM, Cappaert NL, Witter MP (2009). The anatomy of memory: an interactive overview of the parahippocampal-hippocampal network.. Nature Reviews Neuroscience.

[25] Taube JS, Muller RU, Ranck JB (1990). Head-direction cells recorded from the postsubiculum in freely moving rats. I. Description and quantitative analysis.. Journal of Neuroscience.

[26] Boccara CN, Sargolini F, Hult-Thoresen VM, Witter MP, Moser EI (2008). Laminar analysis of grid cells in presubiculum and parasubiculum..

[27] Lengyel M, Szatmáry Z, Érdi P (2003). Dynamically detuned oscillations account for the coupled rate and temporal code of place cell firing.. Hippocampus.

[28] Manwani A, Koch C (1999). Detecting and estimating signals in noisy cable structures: I. Neuronal noise sources.. Neural Computation.

[29] White JA, Rubinstein JT, Kay AR (2000). Channel noise in neurons.. Trends in Neural Systems.

[30] White JA, Haas JS, Moss F, Gielen S (2001). Intrinsic noise from voltage-gated ion channels: effects on dynamics and reliability in intrinsically oscillatory neurons.. Handbook of Biological Physics, Elsevier Press, volume 4.

[31] Verechtchaguina T, Sokolov IM, Schimansky-Geier L (2007). Interspike interval densities of resonate and fire neurons.. BioSystems.

[32] Galán RF, Ermentrout GB, Urban NN (2007). Stochastic dynamics of uncoupled neural oscillators: Fokker-Planck studies with the finite element method.. Physical Review E.

[33] Tahvildari B, Alonso AA (2005). Morphological and electrophysiological properties of lateral entorhinal cortex layers II and III principal neurons.. J Comp Neurol.

[34] Klink R, Alonso A (1993). Ionic mechanisms for the subthreshold oscillations and differential electroresponsiveness of medial entorhinal cortex layer II neurons.. J Neurophysiol.

[35] Klink R, Alonso A (1997). Morphological characteristics of layer II projection neurons in the rat medial entorhinal cortex.. Hippocampus.

[36] Breen TE, Morzorati SL (1996). Innate differences in medial septal area burst firing neurons and the hippocampal theta rhythm during ambulation in selectively bred rat lines.. Brain Res.

[37] Eichenbaum H, Lipton PA (2008). Towards a functional organization of the medial temporal lobe memory system: Role of the parahippocampal and medial entorhinal cortical areas.. Hippocampus.

[38] Mizumori SJY, Williams JD (1993). Directionally selective mnemonic properties of neurons in the lateral dorsal nucleus of the thalamus of rats.. Journal of Neuroscience.

[39] Knierim JJ, Kudrimoti HS, McNaughton BL (1998). Interactions between idiothetic cues and external landmarks in the control of place cells and head direction cells.. Journal of Neurophysiology.

[40] Quirk GJ, Muller RU, Kubie JL (1990). The firing of hippocampal place cells in the dark depends on the rat's recent experience.. Journal of Neuroscience.

[41] Goodridge JP, Dudchenko PA, Worboys KA, Golob EJ, Taube JS (1998). Cue control and head direction cells.. Behavioral Neuroscience.

[42] Burgess N (2008). Grid cells and theta as oscillatory interference: theory and predictions.. Hippocampus.

[43] Fransén E, Tahvildari B, Egorov AV, Hasselmo ME, Alonso AA (2006). Mechanism of graded persistent cellular activity of entorhinal cortex layer V pyramidal neurons.. Neuron.

[44] Jeewajee A, Barry C, O'Keefe J, Burgess N (2008). Grid cells and theta as oscillatory interference: Electrophysiological data from freely moving rats.. Hippocampus.

[45] Barry C, Hayman R, Burgess N, Jeffery KJ (2007). Experience-dependent rescaling of entorhinal grids.. Nat Neurosci.

[46] Vinogradova OS (1995). Expression, control, and probable functional significance of the neuronal theta-rhythm.. Progress in Neurbiology.

[47] Ham D, Hajimiri A (2003). Virtual damping and Einstein relation in oscillators.. IEEE Journal of Solid-State Circuits.

[48] Chow D, Miller M (2005). The relationship between variance of time measurements and the autocorrelation function..

[49] Galán RF, Fourcaud-Trocmé N, Ermentrout GB, Urban NN (2006). Correlation-induced synchronization of oscillations in olfactory bulb neurons.. J Neurosci.

[50] Marella S, Ermentrout GB (2008). Class-II neurons display a higher degree of stochastic synchronization than class-I neurons.. Phys Rev E.

[51] Davie JT, Kole MHP, Rancz EA, Spruston N, Stuart GJ (2006). Dendritic patch-clamp recording.. Nature Protocols.

[52] Langston RF, Ainge JA, Moser EI, Moser MB (2008). Development of grid-like spatial representations in the medial entorhinal cortex of the juvenile rat..

[53] Reboreda A, Raouf R, Alonso A, Séguéla P (2007). Development of cholinergic modulation and graded persistent activity in layer V of medial entorhinal cortex.. Journal of Neurophysiology.

[54] Burton BG, Economo MN, Lee GJ, White JA (2008). Development of theta rhythmicity in entorhinal stellate cells of the juvenile rat.. Journal of Neurophysiology.

