# Supplementary material for: Evaluation of the Oscillatory Interference Model of Grid Cell Firing through Analysis and Measured Period Variance of Some Biological Oscillators
Source: PLoS Comput Biol. 2009 Nov 20;5(11):e1000573. doi: 10.1371/journal.pcbi.1000573 (PMC2773844; doi:10.1371/journal.pcbi.1000573)
Supplement: Text S1 — Technical notes on the 2 VCO convolution (0.06 MB PDF) [file pcbi.1000573.s001.pdf]

Evaluation of the oscillatory interference model of grid cell firing through analysis and measured period variance of some biological oscillators

Eric A. Zilli[1,*], Motoharu Yoshida[1], Babak Tahvildari[2], Lisa M. Giocomo[1,3] Michael E. Hasselmo[1]
1 Department of Psychology, Boston University, Boston, MA 02215, USA.
2 Department of Neurology and Neurosurgery, Montreal Neurological Institute, McGill University, Montreal, Canada. Current address: Department of Neurobiology, School of Medicine, Yale University, New Haven, CT 06520, USA.
3 Current address: Kavli Institute for Systems Neuroscience and Centre for the Biology of Memory, Trondheim NO-7489, Norway.
∗ E-mail: zilli@bu.edu

Text S1 - Technical notes on the 2 VCO convolution

In the main text it was shown numerically that for two VCOs coding two-dimensional positions, an integral over phase shifts due to noise can be calculated as an equivalent spatial convolution. This is of interest because performing convolutions is computationally much more efficient than the slower integral over phase shifts. Here we give a brief analytic result verifying the equivalence and some technical information about calculating the convolution.

Recall in the one VCO case that $g(x, \phi) = g(x - \phi, 0)$ held. We can write the two-dimensional, two-VCO analogue of the function g as $g(x, y, \theta_1, \phi_1, \theta_2, \phi_2)$ where (x, y) is a rectangular coordinate, θ_1 and θ_2 the preferred directions of the two VCOs (these were implicit in the one VCO case), and ϕ_1 and ϕ_2 are phase shifts along the two preferred directions. Letting $R(x)$ be the ramp function to model half-wave rectification ($R(x) = 0$ if $x \leq 0$ and $R(x) = x$ if $x > 0$):

$$
\begin{aligned}
g(x, y, \theta_1, \phi_1, \theta_2, \phi_2) = \frac{1}{2\pi} \int_0^{2\pi} & H[R(\cos(t) + \cos(t + x\cos(\theta_1) + y\sin(\theta_1) - \phi_1)) \\
& \cdot R(\cos(t) + \cos(t + x\cos(\theta_2) + y\sin(\theta_2) - \phi_2)) - \theta] dt
\end{aligned}
$$

If there is always some x' and y' such that $g(x, y, \theta_1, \phi_1, \theta_2, \phi_2) = g(x - x', y - y', \theta_1, 0, \theta_2, 0)$ for any given ϕ_1 and ϕ_2, then integrals over the phase differences can be performed as spatial convolutions. We calculate the x' and y' as follows.

Consider Figure 3 in the main text. Notice that the bands are perpendicular to the directions θ_1 and θ_2, and $\theta_2 - \theta_1 = \pi/3$ (there are other possible angles that would still produce a hexagonal grid, but a specific value is chosen here to fix ideas). As we shift the phase of the bands encoding direction θ_1, we can see in Figure 3 that place fields will slide along the band in the direction $\theta_2 - \pi/2$, and sliding the θ_1 bands by the amount ϕ_1 causes the place fields to move distance $p = \phi_1/\cos(\pi/6) = 2\phi_1/\sqrt{3}$ ($\pi/6$ the angle between θ_1 and $\theta_2 - \pi/2$). If we treat the center of one of the original fields as the location $(0, 0)$, we can find the shifted coordinates of the field by converting and simplifying the polar coordinates with radius $2\phi_1/\sqrt{3}$ and angle $\theta_2 - \pi/2$ to rectangular coordinates $(2\phi_1 \cos(\theta_2 - \pi/2)/\sqrt{3}, 2\phi_1 \sin(\theta_2 - \pi/2)/\sqrt{3})$.

By similar reasoning one can work out that by simultaneously shifting the bands by ϕ_1 and ϕ_2, respectively, the field that was centered at $(0, 0)$ shifts to $(2(\phi_1 \cos(\theta_2 - \pi/2) + \phi_2 \cos(\theta_1 - \pi/2)/\sqrt{3}, 2(\phi_1 \sin(\theta_2 - \pi/2) + \phi_2 \sin(\theta_1 - \pi/2)/\sqrt{3})$. This is analytic confirmation that each phase shift corresponds to a spatial shift.

To calculate the expected population representation for the case of noise in only the two VCOs, we first convolve along the direction of one oscillator's bands with a wrapped normal distribution kernel just as in the one VCO case. A shift in the second oscillator would similarly be modeled as a convolution

along the direction perpendicular to the first VCO's preferred direction. This can be calculated in two passes: first convolving the noise-free spatial pattern along the direction of one band, then convolving the result along the direction of the second band.

To then easily include the effects of noise, a third convolution is needed. This convolution occurs along the direction between the preferred directions of the two VCOs. The size of the kernel must be scaled, however, to account for the greater distance between fields in this direction. Specifically it must be $\sqrt{3}$ larger in both directions. This is true because the wrapped normal distribution is defined over the interval from 0 to 2π, which we have equated with the distance between two neighboring grid fields. However, the nearest field in the direction halfway between the band directions is $2\pi\sqrt{3}$ away and the WN distribution needs to be defined over that whole distance, so it is scaled to be $\sqrt{3}$ times larger.
