# Supplementary material for: Evaluation of the Oscillatory Interference Model of Grid Cell Firing through Analysis and Measured Period Variance of Some Biological Oscillators
Source: PLoS Comput Biol. 2009 Nov 20;5(11):e1000573. doi: 10.1371/journal.pcbi.1000573 (PMC2773844; doi:10.1371/journal.pcbi.1000573)
Supplement: Text S2 — Diffusion analysis (0.09 MB PDF) [file pcbi.1000573.s002.pdf]

Evaluation of the oscillatory interference model of grid cell firing through analysis and measured period variance of some biological oscillators

Eric A. Zilli[1,*], Motoharu Yoshida[1], Babak Tahvildari[2], Lisa M. Giocomo[1,3] Michael E. Hasselmo[1]
1 Department of Psychology, Boston University, Boston, MA 02215, USA.
2 Department of Neurology and Neurosurgery, Montreal Neurological Institute, McGill University, Montreal, Canada. Current address: Department of Neurobiology, School of Medicine, Yale University, New Haven, CT 06520, USA.
3 Current address: Kavli Institute for Systems Neuroscience and Centre for the Biology of Memory, Trondheim NO-7489, Norway.
∗ E-mail: zilli@bu.edu

Text S2 - Diffusion analysis

Burak and Fiete ([1]) measured drift in the encoding of position by examining the square of the displacement of the spatial firing as a function of time. In their continuous attractor model, the spatial firing pattern always remains a perfect hexagonal grid and so the definition of spatial phase is always clear. They found that the square of the displaced distance was proportional to the time interval of the drift, indicative of a diffusive (i.e. random walk) process. They accurately estimated the diffusion coefficient D_{trans} by fitting to the simulated data. They showed $D_{trans} \propto \frac{CV^2}{N}$ where CV is the coefficient of variation of neural spiking (a sub-Poisson process in their model) and N is the number of neurons.

Here we show that the drift in the oscillatory interference model is also diffusive (which is intuitively clear from the noise model alone) and analytically solve for the expected displacement as a function of variance of noise (which is proportional to time, see main text). Using elementary concepts from linear algebra and probability theory, we prove the following theorem.

Theorem. In an oscillatory interference model with n VCOs (equally distributed among preferred directions ϕ_1, ϕ_2, and ϕ_3 which are separated by multiples of $2\pi/3$ and not all co-linear) and cumulative noise variance of σ^2 in both the baseline oscillator and in each of the dendritic oscillators, the expected square of the displacement due to noise $|\hat{c}|^2$ is $E[|\hat{c}|^2] = \frac{4\sigma^2}{n}$. When the preferred directions are instead allowed to be separated by multiples of $\pi/3$ and not all co-linear, the displacement has a more complex form.

As the main text described, variance increases linearly with time, so when the preferred directions are arranged as in the theorem, the square of the displacement is proportional to the amount of time that has passed, just as in the continuous attractor model. Also analogous to the continuous attractor model, the expected displacement is inversely proportional to the number of VCOs (cf. the number of neurons).

Proof. In the first part of the proof, we find the general form relating 2D spatial coordinates c to the corresponding vector of phase differences p and invert this to be able to take a possibly inconsistent vector of phase differences (see next paragraph) and find the coordinate $\hat{c}$ that the inconsistent vector is nearest to. We then relate a probability distribution over all phase differences to the nearest corresponding spatial coordinates and finally simplify the resulting expression to find the expected (mean) spatial coordinates given a distribution of phases.

In the oscillatory interference model, when there are more than two VCOs, noise can cause new fields to appear and existing fields can move slightly so that, given such a spatial pattern, it may not be trivial to define a spatial phase (e.g., see the upper left in Figure 6 in the main text). We solve this problem by computing a least-squares estimator of the encoded spatial phase. That is, only a subset of phase difference vectors actually correspond to real locations, and noise will tend to push the system into regions of the phase-difference space that do not correspond to real locations. Nevertheless, there must always be one real location whose corresponding phase differences are closest to the invalid phase difference vector.

Let the model have $n = 3m$ VCOs for m a positive integer (the result easily generalizes for a greater number of directions, e.g. 6, as is sometimes used). We assume that the VCOs are divided into three groups, each with the same preferred direction spaced in $\pi/3$ intervals. Call the three preferred directions ϕ_1 through ϕ_3. We form a matrix H with two columns and n rows where the first m rows equal $[\cos(\phi_1)\ \ \sin(\phi_1)]$, the second m rows equal $[\cos(\phi_2)\ \ \sin(\phi_2)]$ and the third m equal $[\cos(\phi_3)\ \ \sin(\phi_3)]$. The product Hc for a column 2D coordinate vector c translates the coordinate c into normalized phase differences (a 1 in the vector is a phase difference of 2π). That is, $Hc = p$ is the linear system relating phase vector p and position c.

Because c has 2 elements, when H has more than 2 rows, the system is over-determined and elementary linear algebra tells us that $\hat{c} = (H^T H)^{-1} H^T p$ is the least-squares approximation of the coordinates for an arbitrary p.

We are not given a specific p but we do know the p's distribution. $p = d + b$ is a sum of a common baseline phase difference $b \sim N[0, \sigma^2]$ plus independent phase differences for each VCO $d_i \sim N[0, \sigma^2]$. Thus any pair of elements in p has covariance σ^2 and each element has variance $2\sigma^2$. Let Σ be a covariance matrix expressing this. Then $p \sim MN[0, \Sigma]$ is a multivariate normally distributed vector.

The theorem essentially asks: what is the expected value of $|\hat{c}|^2$ given H as above, $\hat{c} = (H^T H)^{-1} H^T p$ and $p \sim MN[0, \Sigma]$?

We substitute in the value of H and find that

$$(H^T H)^{-1} H^T = \begin{bmatrix} 2\cos(\phi_1)/n & \cdots & 2\cos(\phi_2)/n & \cdots & 2\cos(\phi_3)/n & \cdots \\ 2\sin(\phi_1)/n & \cdots & 2\sin(\phi_2)/n & \cdots & 2\sin(\phi_3)/n & \cdots \end{bmatrix}. \tag{1}$$

The first $m = n/3$ columns are equal, the second m columns are equal, and the third m columns all equal each other, so only one column from each set is shown.

The effect of right multiplying $(H^T H)^{-1} H^T$ by p is to sum the columns, weighting them with random values drawn from $N[0, 2\sigma^2]$ and with covariance σ^2. We can write this as an equation with three terms (one for each set of ten columns), separating the p coefficient into the common baseline and unique VCO phase shifts.

$$\hat{c} = (H^T H)^{-1} H^T p =$$

$$\sum_{i=1}^{m} (d_i + b) \begin{bmatrix} 2\cos(\phi_1)/n \\ 2\sin(\phi_1)/n \end{bmatrix} + \sum_{i=m+1}^{2m} (d_i + b) \begin{bmatrix} 2\cos(\phi_2)/n \\ 2\sin(\phi_2)/n \end{bmatrix} + \sum_{i=2m+1}^{n} (d_i + b) \begin{bmatrix} 2\cos(\phi_3)/n \\ 2\sin(\phi_3)/n \end{bmatrix}$$

$$= \sum_{i=1}^{m} (d_i) \begin{bmatrix} 2\cos(\phi_1)/n \\ 2\sin(\phi_1)/n \end{bmatrix} + \sum_{i=m+1}^{2m} (d_i) \begin{bmatrix} 2\cos(\phi_2)/n \\ 2\sin(\phi_2)/n \end{bmatrix} + \sum_{i=2m+1}^{n} (d_i) \begin{bmatrix} 2\cos(\phi_3)/n \\ 2\sin(\phi_3)/n \end{bmatrix}$$

$$+ (2b/3) \begin{bmatrix} \cos(\phi_1) + \cos(\phi_2) + \cos(\phi_3) \\ \sin(\phi_1) + \sin(\phi_2) + \sin(\phi_3) \end{bmatrix}$$

Notice that when the preferred directions are equally distributed over $(0, 2\pi]$ then the last term equals 0 because the sines and cosines each cancel out. To calculate the general case, this term would need to be carried through the following steps, but we do not do so at present. Notice, though, that this term of displacement due to baseline noise is independent of the number of oscillators (and numerically is of a large magnitude compared to the sum of the other terms when the preferred directions are not equally distributed).

In the first three terms, the column vectors are constants multiplying each $d_i \sim N[0, \sigma^2]$. We can use the property of normally distributed variables that if $X \sim N[\mu, \sigma^2]$ then $aX \sim N[a\mu, (a\sigma)^2]$. Each of these terms can thus be treated as its own pair of random variables and we have $\hat{c}$ distributed as:

$$\hat{c} \sim \sum_{i=1}^{3} \begin{bmatrix} N[0, \frac{n}{3}(\frac{2\sigma\cos(\phi_i)}{n})^2] \\ N[0, \frac{n}{3}(\frac{2\sigma\sin(\phi_i)}{n})^2] \end{bmatrix}$$
$$\sim \begin{bmatrix} N[0, \frac{n}{3}(\frac{4\sigma^2\sum_{i=1}^{3}\cos^2(\phi_i)}{n^2})] \\ N[0, \frac{n}{3}(\frac{4\sigma^2\sum_{i=1}^{3}\sin^2(\phi_i)}{n^2})] \end{bmatrix}$$

The top item in the columns corresponds to the distribution of the x coordinate and the bottom item to the y coordinate. The square of the displacement $|\hat{c}|^2 \sim x^2 + y^2$ is distributed as the sum of the square of the two coordinate distributions. We can simplify the standard deviation of these distributions and pull them out to be coefficients of a distribution with unit variance.

The x and y drift due to VCO noise are distributed as:

$$x \sim \sqrt{\frac{4\sigma^2\sum_{i=1}^{3}\cos^2(\phi_i)}{3n}} N[0,1]$$
$$y \sim \sqrt{\frac{4\sigma^2\sum_{i=1}^{3}\sin^2(\phi_i)}{3n}} N[0,1].$$

Now we simplify the distribution of the sum $x^2 + y^2$.

$$\begin{aligned} x^2 + y^2 &\sim \left(\sqrt{\frac{4\sigma^2\sum_{i=1}^{3}\cos^2(\phi_i)}{3n}} N[0,1]\right)^2 + \left(\sqrt{\frac{4\sigma^2\sum_{i=1}^{3}\sin^2(\phi_i)}{3n}} N[0,1]\right)^2 \\ &\sim \left(\frac{4\sigma^2\sum_{i=1}^{3}\cos^2(\phi_i)}{3n} + \frac{4\sigma^2\sum_{i=1}^{3}\sin^2(\phi_i)}{3n}\right)(N[0,1])^2 \\ &= \left(\frac{4\sigma^2\sum_{i=1}^{3}[\cos^2(\phi_i)+\sin^2(\phi_i)]}{3n}\right)(N[0,1])^2 \\ &= \left(\frac{4\sigma^2}{n}\right)(N[0,1])^2 \end{aligned}$$

Finally, notice that $E[N[0,1]^2] = 1$ because it is a chi-square distribution with df=1. Thus $E[x^2+y^2] = E[|\hat{c}|^2] = \frac{4\sigma^2}{n}$. This concludes the proof.

We can also calculate the variance: $\mathrm{var}(|\hat{c}|^2) = \mathrm{var}\left(\left(\frac{4\sigma^2}{n}\right)(N[0,1])^2\right) = \left(\frac{4\sigma^2}{n}\right)^2 \mathrm{var}(N[0,1]^2) = \frac{32\sigma^4}{n^2}$ (recalling chi-square variance $\mathrm{var}(N[0,1]^2) = 2$).

References

1. Burak Y, Fiete IR (2009) Accurate path integration in continuous attractor network models of grid cells. PLoS Computational Biology 5.
